# SHP465 Mixed Amphetamine Salts in the Treatment of Attention-Deficit/Hyperactivity Disorder in Children and Adolescents: Results of a Randomized, Double-Blind Placebo-Controlled Study

**DOI:** 10.1089/cap.2017.0053

**Published:** 2018-10-20

**Authors:** Matthew Brams, Ann C. Childress, Michael Greenbaum, Ming Yu, Brian Yan, Margo Jaffee, Brigitte Robertson

**Affiliations:** ^1^ Baylor College of Medicine, Houston, Texas.; ^2^ Center for Psychiatry and Behavioral Medicine, Las Vegas, Nevada.; ^3^ Capstone Clinical Research Center, Libertyville, Illinois.; ^4^ Shire, Lexington, Massachusetts.

**Keywords:** adolescents, attention-deficit/hyperactivity disorder, children, SHP465 mixed amphetamine salts

## Abstract

***Objective:*** The aim of this study was to evaluate the efficacy, safety, and tolerability of SHP465 mixed amphetamine salts (MAS) in children and adolescents with attention-deficit/hyperactivity disorder (ADHD).

***Methods:*** This randomized, double-blind dose-optimization study enrolled children and adolescents (6–17 years) meeting *Diagnostic and Statistical Manual of Mental Disorders, Fourth Edition, Text Revision* ADHD criteria and having baseline ADHD Rating Scale IV (ADHD-RS-IV) total scores ≥28. Participants were randomized 1:1 to placebo or dose-optimized SHP465 MAS (12.5–25 mg) for 4 weeks. Total score change (baseline to week 4) on the ADHD-RS-IV (primary endpoint) and the Clinical Global Impressions-Improvement (CGI-I) scale score at week 4 (key secondary endpoint) were assessed using linear mixed-effects models for repeated measures. Safety and tolerability assessments (secondary endpoints) included treatment-emergent adverse events (TEAEs) and vital sign changes.

***Results:*** Of 264 randomized participants (placebo, *n* = 132; SHP465 MAS, *n* = 132), 234 (placebo, *n* = 118; SHP465 MAS, *n* = 116) completed the study. The least squares mean (95% confidence interval) treatment difference significantly favored SHP465 MAS over placebo for ADHD-RS-IV total score change from baseline to week 4 (−9.9 [−13.0, −6.8]; *p* < 0.001; effect size = 0.80) and CGI-I score at week 4 (−0.8 [−1.1, −0.5]; *p* < 0.001; effect size = 0.65). TEAE frequency was 46.6% (61/131) with placebo and 67.4% (89/132) with SHP465 MAS; no serious TEAEs were reported. TEAEs reported at a frequency of ≥5% and ≥2 times the placebo rate were decreased appetite, insomnia, irritability, nausea, and decreased weight. Mean ± standard deviation increases (baseline to final on-treatment assessment) were higher with SHP465 MAS than placebo for pulse (5.7 ± 11.78 vs. 0.7 ± 10.79), systolic blood pressure (3.8 ± 9.15 vs. 2.1 ± 8.72), and diastolic blood pressure (4.0 ± 8.23 vs. 0.5 ± 7.45).

***Conclusions:*** SHP465 MAS demonstrated superiority over placebo in improving ADHD symptoms and global functioning in children and adolescents with ADHD. The safety and tolerability profile of SHP465 MAS was consistent with that of SHP465 MAS in adults and other long-acting psychostimulants in children and adolescents.

## Introduction

Psychostimulants are considered first-line therapies for the treatment of attention-deficit/hyperactivity disorder (ADHD) in children and adolescents (Pliszka 2007; Atkinson and Hollis 2010). Multiple long-acting psychostimulant formulations are available for use in the treatment of children and adolescents with ADHD (Thomas et al. 2013; Briars and Todd 2016). However, prescribers sometimes supplement existing long-acting psychostimulants with an immediate-release psychostimulant later to potentially extend symptom coverage and maintain after-school activities in children and adolescents with ADHD (Briars and Todd 2016). Requiring individuals to take medication multiple times a day can lead to challenges in adherence in individuals with ADHD (Steinhoff 2004), suggesting that once-daily dosing is an important aspect of ADHD pharmacotherapy. In support of the importance of once-daily dosing, in a discrete choice experiment assessing preferred medication attributes, the number of administrations per day was reported as an important attribute in adolescents with ADHD (Glenngard et al. 2013).

SHP465 mixed amphetamine salts (MAS) is a once-daily, extended-release, single-entity MAS product for oral administration approved for use in the United States for the treatment of ADHD in patients 13 years and older. SHP465 MAS contains equal amounts (by weight) of four salts: dextroamphetamine sulfate, amphetamine sulfate, dextroamphetamine saccharate, and amphetamine aspartate monohydrate. This results in a 3:1 mixture of dextro- to levoamphetamine base equivalent. SHP465 MAS capsules contain three types of drug-releasing beads, an immediate-release bead and two different types of delayed-release (DR) beads (Ermer et al. 2007). The first DR bead releases amphetamine at pH 5.5 and the other DR bead releases amphetamine at pH 7.0.

The short-term efficacy, safety, and tolerability of SHP465 MAS in adults with ADHD have been investigated in several phase 3 clinical trials. In a 7-week study, dose-optimized SHP465 MAS (12.5–75 mg) exhibited superiority over placebo in reducing ADHD Rating Scale IV (ADHD-RS-IV) total score (the primary efficacy endpoint) (Spencer et al. 2008). In a 6-week study, forced-dose SHP465 MAS (25, 50, or 75 mg) also produced significantly greater reductions in ADHD-RS-IV total score than placebo (Frick et al. 2017). Across these short-term phase 3 clinical studies, treatment-emergent adverse events (TEAEs) that occurred in ≥10% of participants taking SHP465 MAS were insomnia, dry mouth, decreased appetite, headache, and decreased weight (Spencer et al. 2008; Frick et al. 2017).

To date, there are no published phase 3 clinical studies on the efficacy, safety, and tolerability of SHP465 MAS in children (aged 6–12 years) and adolescents (aged 13–17 years) with ADHD. As such, this report is the first to describe a phase 3 clinical study of SHP465 MAS in children and adolescents with ADHD. The objectives were to evaluate the efficacy, safety, and tolerability of SHP465 MAS versus placebo in the treatment of children and adolescents with ADHD.

## Methods

### Study design and treatment

This was a randomized, multicenter, double-blind, placebo-controlled dose-optimization study (ClinicalTrials.gov: NCT02466425). The study was conducted at 36 sites in the United States between June 18, 2015, and February 16, 2016. The study protocol, protocol amendments, final informed consent document, and relevant supporting information were submitted to and approved by an Institutional Review Board and regulatory agency before study initiation. This study was conducted in accordance with the International Conference on Harmonisation of Good Clinical Practice, principles of the Declaration of Helsinki, and local ethical and legal requirements. Written informed consent was obtained before conducting any study-specific procedures; consent and assent were documented by the dated signature of the participant and the participant's parent(s) or legally authorized representative(s).

The study consisted of four periods: a screening and washout period, a dose-optimization period of 2 weeks, a dose-maintenance period of 2 weeks, and a safety follow-up period. Participants were randomized 1:1 to placebo or dose-optimized SHP465 MAS (12.5 or 25 mg) after the screening and washout period, which allowed for carryover effects of previous medications to abate. Randomization was stratified by age group (children vs. adolescents) to facilitate balance of treatment allocation. Treatment allocation was automatically assigned using interactive response technology; to protect study blinding, placebo capsules were indistinguishable from SHP465 MAS capsules.

The doses chosen for use in this study were determined based primarily on results from an unpublished phase 2 study in adolescents with ADHD. This study evaluated the safety and duration of effect of SHP465 MAS and demonstrated that 25 mg SHP465 MAS in adolescents with ADHD provided the target duration of effect and exhibited an overall safety and tolerability profile that was similar to that seen in other studies of MAS. Although no clinical data were available on the use of SHP465 MAS in children aged 6–12 years at the time the study was designed, it was speculated that the potential benefit–risk ratio of SHP465 MAS in children was similar to that of adolescents. Given the tolerability and efficacy of 25 mg SHP465 MAS in adolescents with ADHD and the relatively lower body weight of children compared with adolescents, 12.5 mg SHP465 MAS was selected as an initial dose in this dose-optimization study.

During dose optimization, all participants randomized to SHP465 MAS initiated treatment at a dose of 12.5 mg. Participants were titrated to 25 mg SHP465 MAS by the investigator, based on symptom response and tolerability, at the week 1 visit. The investigator categorized participant response into one of three conditions (intolerable response, ineffective response, and acceptable response), with acceptable response defined as the participant having achieved ≥30% ADHD-RS-IV total score reduction from baseline, having a Clinical Global Impressions-Improvement (CGI-I) score of 1 (very much improved) or 2 (much improved), and the dose being well tolerated. A single dose reduction was permitted during the dose-optimization period, with the last dose change allowed at the week 2 visit; those unable to tolerate SHP465 MAS were discontinued. During dose maintenance, participants were maintained on the optimized dose of SHP465 MAS established during the dose-optimization period. Throughout the study, study medication was to be taken at 7 AM (±2 hours).

### Participants

Males or nonpregnant nonlactating females (aged 6–17 years at the time of consent) with a primary ADHD diagnosis based on *Diagnostic and Statistical Manual of Mental Disorders, Fourth Edition, Text Revision (DSM-IV-TR)* criteria and baseline ADHD-RS-IV total scores ≥28 were eligible to participate. Participants were also required to have a satisfactory medical assessment with no clinically significant or relevant abnormalities and to be functioning at an age-appropriate intellectual level. Individuals were also required to be untreated or not to be completely satisfied with their current ADHD medication; those who were satisfied with their current treatment were not eligible for study participation.

Key exclusion criteria included having a comorbid psychiatric diagnosis with significant symptoms/symptomatic manifestations that could contraindicate treatment or confound efficacy or safety assessments or a concurrent condition that could confound safety assessments or increase participant risk; a history of a chronic tic disorder, a current tic disorder, a history of tics that were judged by the investigator to be exclusionary, or a diagnosis of Tourette's syndrome; being considered a suicide risk, or having had a previous suicide attempt, or demonstrating active suicidal ideation; being underweight (body mass index [BMI] <3rd percentile) or overweight (BMI >97th percentile) at screening based on the Centers for Disease Control and Prevention's age- and sex-specific values; having blood pressure exceeding the 90th percentile for age, sex, and height at screening and baseline; having hypertension; having symptomatic cardiovascular disease, cardiac issues, a clinically significant electrocardiogram (ECG), or a family history of sudden cardiac death or ventricular arrhythmia; having a documented allergy, hypersensitivity, or intolerance to amphetamine or excipients in SHP465 MAS; having failed to respond to a previous course of amphetamine therapy; having a history of suspected substance abuse or dependence (excluding nicotine) based on *DSM-IV-TR* criteria; or taking a prohibited medication that was excluded or had not been appropriately washed out.

### Efficacy endpoints

Efficacy assessments were completed by clinicians experienced in the evaluation of ADHD in children and adolescents. The primary efficacy endpoint was change in ADHD-RS-IV total score from baseline to week 4. The ADHD-RS-IV was developed to assess ADHD symptoms in children and adolescents based on *DSM-IV-TR* criteria (Dupaul et al. 1998). It consists of 18 items designed to reflect current ADHD symptoms, with items scored on 4-point scales (0 [no symptoms] to 3 [severe symptoms]). Total scores range from 0 to 54, with higher scores indicating more severe symptoms. Subscale scores can also be calculated by summing responses to the even-numbered items (hyperactivity/impulsivity subscale) or odd-numbered items (inattentiveness subscale). The ADHD-RS-IV was administered at baseline and all postbaseline visits through week 4/early termination (ET).

The key secondary endpoint was the score on the CGI-I scale at week 4. The CGI-I measures global functioning improvement on a 7-point scale (1 [very much improved] to 7 [very much worse]; 0 indicates not assessed) (Guy 1976), with improvement measured against baseline CGI-Severity (CGI-S) scores. Other secondary efficacy assessments included ADHD-RS-IV hyperactivity/impulsivity and inattentiveness subscale score changes from baseline to week 4 and global functioning improvement based on the dichotomized CGI-I.

### Safety and tolerability endpoints

Adverse events (AEs) were collected from the time of informed consent and at every study visit until the defined follow-up period. TEAEs were defined as AEs that started after the first study drug dose or that started before the first study drug dose, but increased in severity after the first study drug dose. TEAEs were categorized based on severity, relatedness to treatment, and seriousness.

Vital signs and weight were assessed at all visits. Vital signs were measured after ∼3 minutes of rest while the participant was seated and consisted of three assessments taken at ∼2-minute intervals. The 12-lead ECG was assessed at screening, baseline, week 2, and week 4/ET after 3 minutes of rest; the baseline assessment consisted of three recordings taken at 3-minute intervals.

The Columbia-Suicide Severity Rating Scale (C-SSRS) (Posner et al. 2011) was used at baseline and each study visit by an individual who was medically responsible for the participant. The C-SSRS is a semistructured interview designed to capture the occurrence, severity, and frequency of suicide-related thoughts and behaviors.

### Data presentation and statistical analyses

Sample size was determined using nQuery Advisor 7.0 (Statistical Solutions, Ltd., Cork, Ireland). To detect an assumed treatment difference (SHP465 MAS–placebo) of 6.0, with an assumed common standard deviation (SD) of 10.0 for ADHD-RS-IV total score change, 60 participants/group were required to provide 90% power for a two-sided *t*-test (α = 0.05). Taking into account an expected 20% postrandomization dropout rate, at least 150 participants needed to be randomized. A blinded interim sample size reestimation was performed when ∼75% of randomized participants had either completed or discontinued from the study, among which 118 were in the full analysis set (FAS; randomized participants taking ≥1 study drug dose and having ≥1 postdose primary efficacy assessment) and 107 had completed the study. Based on the pooled SD of 13.66, the recalculated sample size was 110 participants/group (220 in total) without changing the original statistical assumptions (90% power; treatment difference of 6.0; two-sided significance level of 0.05). Taking into account an expected 20% postrandomization dropout rate for those not in the interim cohort, the overall randomization target was 264.

Efficacy analyses were performed in the FAS; all statistical tests were two-sided and performed at the 0.05 level of significance. The primary efficacy endpoint, change in ADHD-RS-IV total score from baseline to week 4, was analyzed using the linear mixed-effects model for repeated measures (MMRM) with treatment, visit, age group (6–12 years vs. 13–17 years), and the interaction of treatment × visit as factors; baseline ADHD-RS-IV total score as a covariate; and the interaction of baseline ADHD-RS-IV total score × visit adjusted in the model. Treatment comparisons were based on least squares (LS) mean treatment differences (SHP465 MAS–placebo), with the corresponding 95% confidence interval (CI) and *p*-value; effect size was also determined. Two sensitivity analyses (placebo multiple imputation and multiple imputations with penalties applied to dropouts) were used to examine the robustness of the primary MMRM analysis. Change from baseline at week 4 on the ADHD-RS-IV subscales was also analyzed using the MMRM model described for the primary efficacy endpoint.

Assessment of the key secondary efficacy endpoint, CGI-I score at week 4, was assessed using the MMRM method described for the primary efficacy analysis. The analysis accounted for baseline CGI-S as a covariate and adjusted for the interaction of baseline CGI-S × visit. A supportive analysis of the CGI-I was based on dichotomized improvement (improved vs. not improved) at the final on-treatment assessment. Participants were categorized as improved if the CGI-I score was 1 (very much improved) or 2 (much improved) and as not improved if the CGI-I score was 3 (minimally improved) through 7 (very much worse). Statistical assessment of the dichotomized CGI-I consisted of a Cochran-Mantel-Haenszel (CMH) test stratified by age group and baseline CGI-S.

To preserve study-wide type I error (two-sided α = 0.05) across the primary and key secondary efficacy endpoints, a fixed-sequence test procedure was applied. For this procedure, hypotheses were tested in order (first the primary endpoint and then the secondary endpoint). The secondary endpoint could only attain significance if the primary endpoint was significant at the two-sided 0.05 significance level. For analyses not included in the fixed-sequence test procedure (dichotomized CGI-I and ADHD-RS-IV subscale score changes), reported *p*-values are nominal (unadjusted) and are reported for descriptive purposes only.

Safety and tolerability were analyzed in the safety analysis set (randomized participants taking ≥1 study drug dose). All safety and tolerability data are presented using descriptive statistics. For each endpoint, baseline was assessed as the last value collected before the first dose of the double-blind study drug and the final on-treatment assessment was defined as the last valid assessment obtained after baseline.

## Results

### Participant disposition and demographics

**FIG. 1.**
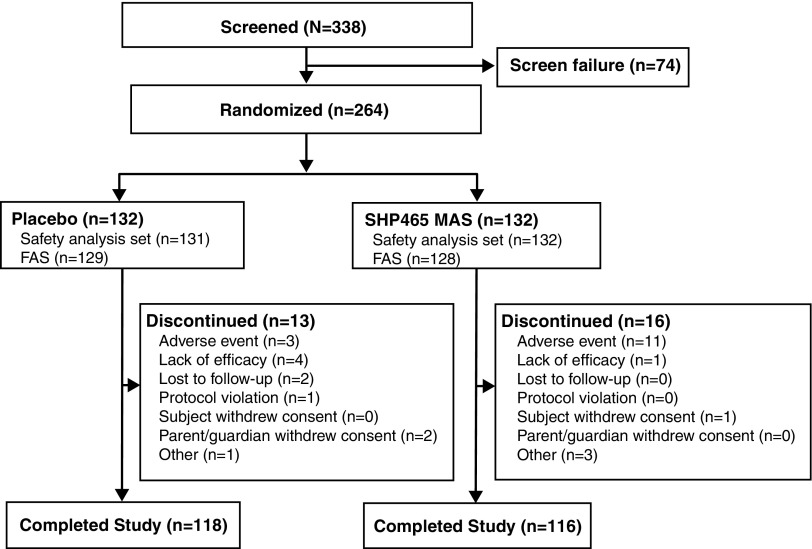
Participant disposition. FAS, full analysis set; MAS, mixed amphetamine salts.

Participant disposition is summarized in Figure 1. Of 338 screened participants, 264 were randomized (placebo, *n* = 132; SHP465 MAS, *n* = 132). During the study, 29 participants (placebo, *n* = 13; SHP465 MAS, *n* = 16) discontinued from the study. The most frequent reason for study discontinuation was lack of efficacy with placebo (*n* = 4) and TEAEs with SHP465 MAS (*n* = 11; see footnote “†” of Table 2 for a complete list of TEAEs leading to study discontinuation). Additional reasons for study discontinuation in ≥2 participants in either treatment group included AEs (placebo, *n* = 3), lost to follow-up (placebo, *n* = 2), withdrawn by parent/guardian (placebo, *n* = 2), and other (SHP465 MAS, *n* = 3).

Demographic and baseline clinical characteristics by treatment group are summarized in Table 1. Most participants were males (163/263 [62.0%]) and white (161/263 [61.2%]); mean ± SD age was 12.5 ± 3.24 years. In the overall study population, mean ± SD time since ADHD diagnosis was 5.0 ± 4.02 years, and most participants (199/263 [75.7%]) were diagnosed as having the combined ADHD subtype.

**Table 1.** Participant Demographics and Clinical Characteristics, Safety Analysis Set

	*Placebo (*n* = 131)*	*SHP465 MAS (*n* = 132)*
Mean ± SD age, years	12.5 ± 3.24	12.4 ± 3.25
Age category, *n* (%)		
6–12 years	52 (39.7)	54 (40.9)
13–17 years	79 (60.3)	78 (59.1)
Gender, *n* (%)		
Male	77 (58.8)	86 (65.2)
Race, *n* (%)		
White	83 (63.4)	78 (59.1)
Black	37 (28.2)	38 (28.8)
Asian	0	1 (0.8)
Multiple	9 (6.9)	12 (9.1)
Other	2 (1.5)	3 (2.3)
Mean ± SD weight, kg	51.92 ± 18.255	51.30 ± 17.560
Mean ± SD BMI, kg/m^2^	20.98 ± 4.143	20.45 ± 3.784
Mean ± SD time since ADHD diagnosis, years	4.9 ± 3.94	5.1 ± 4.11
ADHD subtype, *n* (%)		
Inattentive	29 (22.1)	32 (24.2)
Hyperactive/impulsive	1 (0.8)	2 (1.5)
Combined	101 (77.1)	98 (74.2)
Mean ± SD ADHD-RS-IV score		
Total	40.1 ± 7.01	39.0 ± 7.02
Hyperactivity/impulsivity^*^	17.9 ± 5.52	17.0 ± 5.86
Inattentiveness^*^	22.1 ± 3.57	22.0 ± 3.20
CGI-S category,^†^*n* (%)		
Mildly ill	0	3 (2.3)
Moderately ill	55 (42.0)	53 (40.2)
Markedly ill	64 (48.9)	65 (49.2)
Severely ill	12 (9.2)	11 (8.3)

^*^ Based on full analysis set (*n* = 129 for placebo; *n* = 128 for SHP465 MAS).

^†^ No participants were categorized as normal (not at all ill), borderline mentally ill, or among the most extremely ill.

ADHD, attention-deficit/hyperactivity disorder; ADHD-RS-IV, ADHD Rating Scale IV; BMI, body mass index; CGI-S, Clinical Global Impressions-Severity; MAS, mixed amphetamine salts; SD, standard deviation.

### Prior and concomitant ADHD medications

Prior ADHD medication use was reported by 74.0% (97/131) of the placebo group and 79.5% (105/132) of the SHP465 MAS group. The ADHD medications used most frequently before entering the study were methylphenidate, lisdexamfetamine, and immediate-release MAS (Supplementary Table S1; Supplementary Data are available online at www.liebertpub.com/cap). A concomitant medication was used by 55.0% (72/131) and 53.0% (70/132), respectively, of participants in the placebo and SHP465 MAS groups. The only concomitant ADHD medications used by ≥2% of study participants in either treatment group were lisdexamfetamine, immediate-release MAS, and methylphenidate (Supplementary Table S1); these instances were defined as protocol violations and the number of participants taking these medications was comparable across treatment groups.

### Drug exposure

The mean ± SD duration of exposure was 3.76 ± 0.791 weeks with placebo and 3.77 ± 0.786 weeks with SHP465 MAS. The maximum dose of SHP465 MAS (i.e., the highest dose taken by the participant) was 25 mg in 81.1% (107/132) of participants; only 3.7% (*n* = 4) of these participants were downtitrated to a final dose of 12.5 mg SHP465 MAS. The optimal dose of SHP465 MAS (i.e., the dose at which a participant was judged by the investigator to have attained an acceptable treatment response) was 25 mg in 72.0% (95/132) of participants and 12.5 mg in 24.2% (32/132) of participants. The mean ± SD adherence rate during the double-blind treatment period ([the number of capsules dispensed minus the number of capsules returned]/[the date the bottle was dispensed of following the visit–the date the bottle was dispensed]) was 99.39% ± 14.127% with placebo and 99.67% ± 13.351% with SHP465 MAS.

### Efficacy

**FIG. 2.**
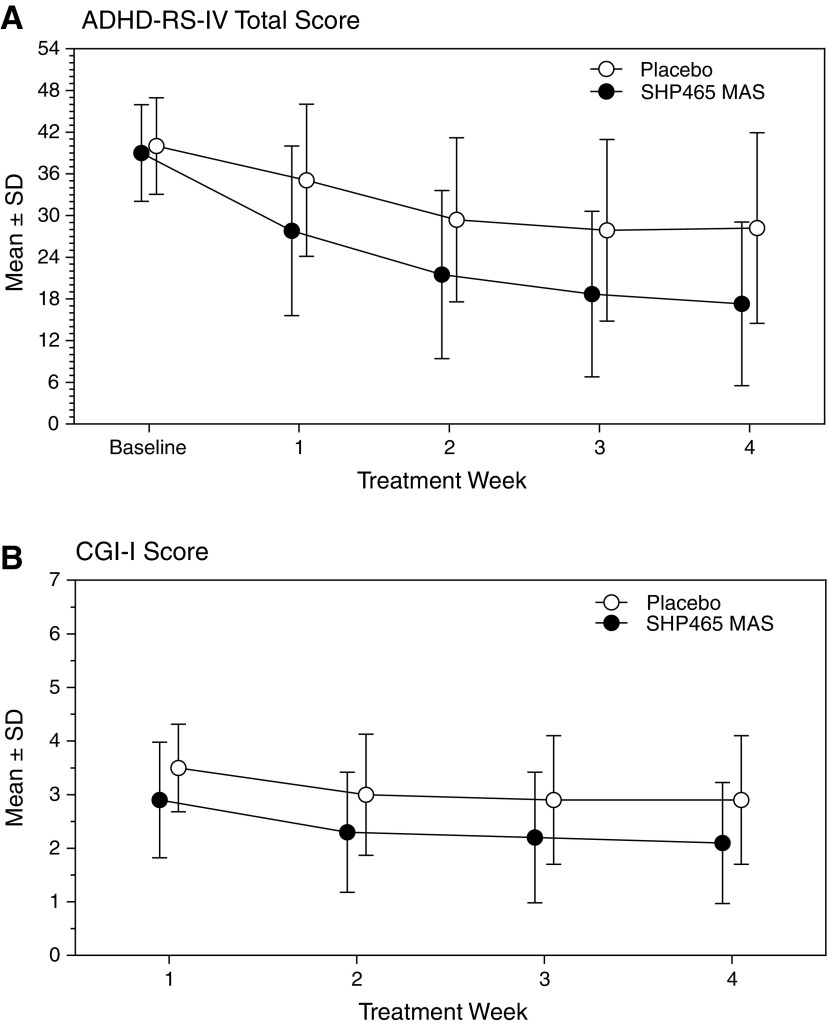
Mean ± SD ADHD-RS-IV total score **(A)** and CGI-I score **(B)** by treatment week, full analysis set. ADHD-RS-IV, ADHD Rating Scale IV; CGI-I, Clinical Global Impressions-Improvement; MAS, mixed amphetamine salts; SD, standard deviation.

Mean ADHD-RS-IV total score decreased in both treatment groups during the course of the study (Fig. 2A; Supplementary Table S2). The LS mean (95% CI) change in ADHD-RS-IV total score from baseline to week 4 was −10.8 (−13.0, −8.5) with placebo and −20.7 (−22.9, −18.5) with SHP465 MAS. The LS mean (95% CI) treatment difference for the change from baseline in ADHD-RS-IV total score at week 4 significantly favored SHP465 MAS over placebo (−9.9 [−13.0, −6.8]; degrees of freedom = 241, *t*-statistic = −6.23, *p* < 0.001; effect size = 0.80). Sensitivity analyses were supportive of the primary MMRM efficacy analysis.

Mean CGI-I score decreased in both treatment groups from weeks 1 to 4 (Fig. 2B; Supplementary Table S2). The LS mean (95% CI) CGI-I score at week 4 was 3.0 (2.8, 3.2) with placebo and 2.2 (2.0, 2.4) with SHP465 MAS. The LS mean (95% CI) treatment difference for CGI-I score at week 4 significantly favored SHP465 MAS over placebo (−0.8 [−1.1, −0.5]; degrees of freedom = 242, *t*-statistic = −5.10, *p* < 0.001; effect size = 0.65).

The LS mean (95% CI) treatment differences for the change from baseline at week 4 on the ADHD-RS-IV hyperactivity/impulsivity and inattentiveness subscales favored SHP465 MAS over placebo (both nominal *p* < 0.001; Supplementary Table S2). On the dichotomized CGI-I, the percentage of participants categorized as improved at the final on-treatment assessment was greater with SHP465 MAS than with placebo (nominal *p* < 0.001 based on CMH test; Supplementary Table S2).

### Safety/tolerability

The frequency of TEAEs was greater with SHP465 MAS than with placebo (Table 3). Most TEAEs were of mild or moderate severity; there were no on-treatment serious TEAEs reported during the study. There were two nonfatal serious AEs of major depression and suicide attempt in one participant randomized to SHP465 MAS; these AEs occurred 7 days after the final study drug dose. This female participant experienced a TEAE of depressed mood (judged by the investigator to be of mild intensity, not serious, and related to the study drug) after 11 days of treatment, which resulted in study discontinuation. During the follow-up period (7 days after discontinuation of the study drug), the participant experienced serious AEs of major depression (moderate intensity) and suicide attempt (severe intensity). Both serious AEs were not considered to be treatment emergent (they occurred >3 days after cessation of treatment) and were judged by the investigator to be not related to the study drug based on communications with the participant's parent.

The frequency of TEAEs leading to discontinuation was greater with SHP465 MAS than with placebo. The TEAEs that led to discontinuation were right bundle branch block, acute psychosis, and suicidal ideation in the placebo group (*n* = 1 for each) and upper abdominal pain, viral infection, increased heart rate, decreased appetite, seizure, depressed mood, insomnia, irritability, hemoptysis, and dizziness in the SHP465 MAS group (*n* = 1 for each, except for dizziness, which occurred in two participants) (see footnote “†” of Table 2). All TEAEs that led to study discontinuation were reported to be of mild to moderate severity, none were considered to be serious by the study investigator, and only two were not considered to be related to the study drug by the investigator (instances of viral infection and seizure in participants randomized to SHP465 MAS); all TEAEs resolved following discontinuation.

**Table 2.** Summary of Safety and Tolerability Endpoints, Safety Analysis Set

	*Placebo (*n* = 131)*	*SHP465 MAS (*n* = 132)*
Any TEAE, *n* (%)	61 (46.6)	89 (67.4)
TEAEs related to the study drug	34 (26.0)	70 (53.0)
Severe TEAEs^*^	1 (0.8)	4 (3.0)
TEAEs leading to discontinuation^†^	3 (2.3)	11 (8.3)
Serious TEAEs	0	0
TEAEs in ≥2% of participants in either treatment group, *n* (%)		
Decreased appetite	9 (6.9)	40 (30.3)
Insomnia^‡^	3 (2.3)	22 (16.7)
Headache	14 (10.7)	16 (12.1)
Irritability	2 (1.5)	9 (6.8)
Nausea	4 (3.1)	9 (6.8)
Weight decreased	1 (0.8)	7 (5.3)
Dizziness	0	6 (4.5)
Heart rate increased	1 (0.8)	5 (3.8)
Abdominal pain upper	2 (1.5)	4 (3.0)
Upper respiratory tract infection	2 (1.5)	3 (2.3)
Fatigue	4 (3.1)	2 (1.5)
Nasopharyngitis	4 (3.1)	0
Oropharyngeal pain	3 (2.3)	0
Weight increased	3 (2.3)	0
Vital signs, mean ± SD change at final on-treatment assessment^¶^		
SBP, mmHg	2.1 ± 8.72	3.8 ± 9.15
DBP, mmHg	0.5 ± 7.45	4.0 ± 8.23
Pulse, bpm	0.7 ± 10.79	5.7 ± 11.78
Vital sign outliers at any time during treatment,^§^*n* (%)		
SBP >120 mmHg and increase of >10 mmHg from baseline in children	3 (6.0)	4 (7.8)
SBP >140 mmHg and increase of >10 mmHg from baseline in adolescents	0	1 (1.3)
DBP >80 mmHg and increase of >10 mmHg from baseline in children	3 (6.0)	3 (5.9)
DBP >90 mmHg and increase of >10 mmHg from baseline in adolescents	0	1 (1.3)
Pulse rate ≥100 bpm and increase >15 bpm from baseline in children	1 (2.0)	9 (17.6)
Pulse rate ≥100 bpm and increase >15 bpm from baseline in adolescents	2 (2.5)	5 (6.5)
Weight at final on-treatment assessment^¶^		
Mean ± SD change at final on-treatment assessment, kg	0.70 ± 1.256	−0.92 ± 1.480
Mean ± SD *z*-score	0.04 ± 0.138	−0.12 ± 0.164
Median *z*-score	0.04	−0.11
Weight decrease ≥7% from baseline, *n* (%)	2 (1.6)	7 (5.5)
BMI at final on-treatment assessment^¶^		
Mean ± SD change at final on-treatment assessment, kg/m^2^	0.30 ± 0.541	−0.37 ± 0.576
Mean ± SD *z*-score	0.08 ± 0.185	−0.14 ± 0.251
Median *z*-score	0.06	−0.10

^*^ Severe TEAEs: placebo (muscle strain [*n* = 1]); SHP465 MAS (nausea [*n* = 1]; back pain [*n* = 1]; neck pain [*n* = 1]; dizziness [*n* = 1]; and lethargy [*n* = 1]; insomnia [*n* = 1]).

^†^ TEAEs leading to discontinuation: placebo (bundle branch block right [*n* = 1]; acute psychosis [*n* = 1]; and suicidal ideation [*n* = 1]); SHP465 MAS (abdominal pain upper [*n* = 1]; viral infection [*n* = 1]; heart rate increased [*n* = 1]; decreased appetite [*n* = 1]; dizziness [*n* = 2]; seizure [*n* = 1]; depressed mood [*n* = 1]; insomnia [*n* = 1]; irritability [*n* = 1]; and hemoptysis [*n* = 1]).

^‡^ Includes preferred terms of insomnia, initial insomnia, and middle insomnia.

^¶^ Based on *n* = 129 for placebo and *n* = 128 for SHP465 MAS.

^§^ Based on *n* = 50 children and *n* = 79 adolescents for placebo, and *n* = 51 children and *n* = 77 adolescents for SHP465 MAS.

BMI, body mass index; DBP, diastolic blood pressure; MAS, mixed amphetamine salts; SBP, systolic blood pressure; SD, standard deviation; TEAE, treatment-emergent adverse event.

The most frequently reported TEAEs (those reported by ≥2% of participants) in the SHP465 MAS treatment arm were decreased appetite, insomnia, headache, irritability, nausea, weight decreased, dizziness, heart rate increased, upper abdominal pain, and upper respiratory tract infection (Table 2). Decreased appetite and insomnia were the most frequently reported TEAEs with SHP465 MAS. The majority of instances of TEAEs related to decreased appetite or insomnia were of mild or moderate intensity, resolved while on the study drug, and did not result in a dose change (Table 3). The mean number of events per individual, mean onset day of events, and mean event duration while on the study drug were roughly comparable for both TEAE types across treatments, with the exception of onset day for decreased appetite that tended to be lower with SHP465 MAS than with placebo (Table 3).

**Table 3.** Summary of Insomnia-Related and Decreased Appetite-Related Treatment-Emergent Adverse Events, Safety Analysis Set

	Insomnia^*^		*Decreased appetite*	
	*Placebo (*n* = 131)*	*SHP465 MAS (*n* = 132)*	*Placebo (*n* = 131)*	*SHP465 MAS (*n* = 132)*
Participants with event, *n* (%)	3 (2.3)	22 (16.7)	9 (6.9)	40 (30.5)
Number of events	3	27	9	44
Mean ± SD number of events per individual^†^	1.0 ± 0	1.2 ± 0.53	1.0 ± 0	1.1 ± 0.38
Mean ± SD onset day of event^‡^	6.3 ± 9.24	6.8 ± 6.36	6.4 ± 4.28	4.4 ± 4.52
Mean ± SD duration of event while on the study drug in days^†^	13.3 ± 2.89	12.1 ± 8.53	19.4 ± 7.58	19.8 ± 9.50
Severity of event, *n* (%)				
Mild	2 (66.7)	14 (51.9)	6 (66.7)	26 (59.1)
Moderate	1 (33.3)	12 (44.4)	3 (33.3)	18 (40.9)
Severe	0	1 (3.7)	0	0
Outcome of event, *n* (%)				
Resolved^§^	3 (100)	26 (96.3)	8 (88.9)	38 (86.4)
Ongoing^¶^	0	1 (3.7)	1 (11.1)	5 (11.6)
Dose adjustment, *n* (%)^£^				
Dose increased	0	0	0	0
Dose reduced	0	1 (3.7)	0	1 (2.3)
Dose not changed	3 (100)	23 (85.2)	8 (88.9)	42 (95.5)
Dose interrupted	0	0	0	0
Dose withdrawn	0	1 (3.7)	0	1 (2.3)
Not applicable	0	2 (7.4)	1 (11.1)	0
Led to discontinuation	0	1 (3.7)	0	1 (2.3)

^*^ Includes adverse events with preferred terms insomnia, initial insomnia, and middle insomnia.

^†^ Overlapping events with different preferred terms were counted as multiple events. If a participant had multiple events of the same type that were not overlapping or adjacent in time, the durations were averaged and summary statistics were based on average durations. Event duration was the number of days from the onset of the event while on the study drug until the earlier of the end date of the event or the date of the last dose +3 days. If the date of the last dose was missing, the date of the last day of study was used. Events that either overlapped or were adjacent in time were merged into one event only when calculating event duration.

^‡^ Calculated as follows: (onset day of first event–date of first dose) +1.

^§^ Events having a resolution date on or before the participant's last dose date +3 days; if the last event of overlapping or adjacent adverse events of the same type was ongoing, the other event was not counted as resolved even if it had a resolution date.

^¶^ Includes adverse events without a resolution date; percentages are based on the number of treatment-emergent events, excluding from the denominator overlapping or adjacent events where a later event was still ongoing.

^£^ Percentages based on the number of treatment-emergent adverse events.

MAS, mixed amphetamine salts; SD, standard deviation.

No participant in the SHP465 MAS group experienced a psychiatric TEAE that was deemed to be of special interest (e.g., psychosis/mania, suicidal events, aggression, and other events); three participants in the placebo group experienced psychiatric TEAEs deemed to be of special interest (psychosis/mania, *n* = 1; suicidal events, *n* = 2).

Mean increases in pulse, diastolic blood pressure, and systolic blood pressure in participants treated with SHP465 MAS were observed (Table 2). The frequency of potentially clinically significant outliers at any time during treatment in pulse and blood pressure was greater with SHP465 MAS than with placebo (Table 2). On the ECG, the mean ± SD change from baseline in the Fridericia-corrected QT (QTcF) interval was −2.9 ± 13.11 msec with placebo and 0.3 ± 13.83 msec with SHP465 MAS at the final on-treatment assessment. A QTcF interval ≥500 msec or an increase in QTcF interval of ≥60 msec was not observed in any participant.

Mean increases in weight and BMI were observed with placebo, whereas mean decreases were observed with SHP465 MAS at the final on-treatment assessment (Table 2). The frequency of weight loss of ≥7% from baseline was greater with SHP465 MAS than with placebo (Table 2).

In the C-SSRS assessment, one participant in the placebo group responded yes to the nonspecific activity suicide thoughts item within the suicidal ideation category. Another participant in the placebo group responded yes to the nonsuicidal self-injurious behavior item of the suicidal behavior category of the C-SSRS; the behavior was not considered suicidal; therefore, the participant was allowed to continue in the study.

## Discussion

In this phase 3 dose-optimization study, SHP465 MAS demonstrated superiority over placebo in the treatment of children and adolescents with ADHD, as measured by the primary efficacy endpoint (reductions in ADHD-RS-IV total score) and the key secondary endpoint (CGI-I score). The superiority of SHP465 MAS was supported by sensitivity analyses for ADHD-RS-IV total score and CGI-I score and by improvement on other secondary efficacy endpoints (ADHD-RS-IV subscale score reductions and dichotomized improvement on the CGI-I). These findings suggest that SHP465 MAS, which contains three types of drug-releasing beads—an immediate-release bead and two different types of DR beads, is an efficacious treatment option in children and adolescents with ADHD. The efficacy of SHP465 MAS in children and adolescents with ADHD that was observed in this study is consistent with findings in adults with ADHD (Spencer et al. 2008; Frick et al. 2017) and for other long-acting psychostimulants in children and adolescents with ADHD (Spencer et al. 2006; Maneeton et al. 2015; Storebo et al. 2015).

The magnitude of treatment effect with SHP465 MAS observed in this study cannot be directly compared with psychostimulant effects reported in other studies due to differences in study design (including patient enrollment criteria, drug dose, and treatment length). However, the magnitude of ADHD-RS-IV total score reductions with SHP465 MAS in this study, which occurred after 2 weeks of dose maintenance, was within a range observed for other long-acting psychostimulants from individual studies (Spencer et al. 2006; Biederman et al. 2007; Newcorn et al. 2008; Findling et al. 2011; Stein et al. 2011; Coghill et al. 2013) and the treatment difference versus placebo was within ranges reported in systematic reviews and/or meta-analyses (Maneeton et al. 2015; Storebo et al. 2015).

The overall safety and tolerability profile of SHP465 MAS in this study was consistent with that of SHP465 MAS in adults with ADHD (Spencer et al. 2008; Frick et al. 2017) and with that of other long-acting psychostimulants in children and adolescents (Biederman et al. 2002; Spencer et al. 2006; Newcorn et al. 2008; Findling et al. 2011; Coghill et al. 2013). The most frequently reported TEAEs (those reported by ≥2% of participants in the SHP465 MAS treatment arm) were decreased appetite, headache, insomnia, irritability, nausea, weight decreased, dizziness, increased heart rate increased, upper abdominal pain, and upper respiratory tract infection. This pattern is consistent with reports on adults with ADHD treated with SHP465 MAS (Spencer et al. 2008; Frick et al. 2017). These TEAEs have also been reported in children and/or adolescents treated with other long-acting psychostimulants (Biederman et al. 2002; Spencer et al. 2006; Newcorn et al. 2008; Findling et al. 2011; Coghill et al. 2013).

The two most frequently reported TEAEs that occurred in participants randomized to SHP465 MAS, decreased appetite and insomnia, are also among the most frequently reported TEAEs in children and/or adolescents treated with other long-acting psychostimulants (Biederman et al. 2002, 2007; Spencer et al. 2006; Newcorn et al. 2008; Findling et al. 2011; Coghill et al. 2013). The frequency of decreased appetite/anorexia as a TEAE has been reported to range from 27.3% to 49.3% with lisdexamfetamine (Biederman et al. 2007; Findling et al. 2011; Coghill et al. 2013), from 17% to 20.7% with osmotic controlled-release oral delivery system (OROS) methylphenidate (Newcorn et al. 2008; Coghill et al. 2013), and from 21.9% to 44.8% with extended-release MAS (Biederman et al. 2002; Spencer et al. 2006). Rates of insomnia-related TEAEs have been reported to range from 9% to 24.7% with lisdexamfetamine (Biederman et al. 2007; Findling et al. 2011; Coghill et al. 2013), from 13% to 14.4% with OROS methylphenidate (Newcorn et al. 2008; Coghill et al. 2013), and from 8.9% to 17.5% with extended-release MAS (Biederman et al. 2002; Spencer et al. 2006).

It is noteworthy that the overall frequency of insomnia as a TEAE was lower in this study (16.7% overall) than in the two SHP465 MAS studies on adults with ADHD (41.7% and 29.2% overall) (Spencer et al. 2008; Frick et al. 2017), possibly because of the lower SHP465 MAS doses used in this study. Compared with placebo, SHP465 MAS was associated with increases in pulse and blood pressure and with decreases in body weight. A similar pattern of changes in vital signs and weight has been reported with SHP465 MAS in adults with ADHD (Spencer et al. 2008; Frick et al. 2017) and with other long-acting psychostimulants in children and/or adolescents with ADHD (Spencer et al. 2006; Newcorn et al. 2008; Findling et al. 2011; Coghill et al. 2013).

### Limitations

The study population was primarily male and white. As such, it is unknown how these data would generalize to a more diverse treatment population. Although the study was not powered to compare subgroup differences, exploratory analyses suggested that the primary efficacy findings by race and gender were generally in agreement with the overall results. Findings for the secondary efficacy endpoints (dichotomized CGI-I and ADHD-RS-IV subscales) and from the exploratory analyses should be interpreted in light of the fact that they were not included in the testing hierarchy. Therefore, the reported *p*-values for these endpoints are nominal (unadjusted) and provided for descriptive purposes only. In addition, the long-term safety and tolerability of SHP465 MAS cannot be determined from these data given the short 4-week duration of the study.

## Conclusions

SHP465 MAS demonstrated superiority over placebo in improving ADHD core symptoms and global functioning in children and adolescents with ADHD. The safety and tolerability profile of SHP465 MAS was similar to that observed in adults and of other long-acting psychostimulants. Based on this study, the SHP465 MAS starting dose in pediatric patients (aged 13–17 years) is suggested to be 12.5 mg once daily in the morning. The dosage may be adjusted in increments of 12.5 mg no sooner than weekly up to the maximum dose of 25 mg per day based on efficacy, tolerability, and safety. SHP465 MAS is not approved for use in children aged 12 years and younger.

## Clinical Significance

Some prescribers may supplement a long-acting psychostimulant with an immediate-release short-acting psychostimulant later in the day for children and adolescents with attention-deficit/hyperactivity disorder (ADHD). Therefore, there is a need for once-daily psychostimulant medications. SHP465 mixed amphetamine salts (MAS) is a long-acting psychostimulant for the treatment of ADHD. The findings of this study indicate that SHP465 MAS is superior over placebo in improving ADHD symptoms and global functioning in children and adolescents with ADHD, with a safety and tolerability profile consistent with that of other long-acting psychostimulants.

## Supplementary Material

Supplemental data

Supplemental data
