# Supplementary material for: SHP465 Mixed Amphetamine Salts in the Treatment of Attention-Deficit/Hyperactivity Disorder in Children and Adolescents: Results of a Randomized, Double-Blind Placebo-Controlled Study
Source: J Child Adolesc Psychopharmacol. 2018 Oct 20;28(1):19–28. doi: 10.1089/cap.2017.0053 (PMC5771539; doi:10.1089/cap.2017.0053)
Supplement: Supplemental data [file Supp_Table1.pdf]

Supplementary Data

Supplementary Table S1. Prior and Concomitant Attention-Deficit/Hyperactivity Disorder Medications, Safety Analysis Set

	Placebo (n = *131)*	*SHP465 MAS* (n = *132)*
Prior ADHD medications used by ≥2% of participants (either treatment arm), *n* (%)		
Methylphenidate	61 (46.6)	61 (46.2)
Lisdexamfetamine	40 (30.5)	42 (31.8)
Immediate-release MAS	40 (30.5)	33 (25.0)
Dexmethylphenidate hydrochloride	12 (9.2)	19 (14.4)
Guanfacine	14 (10.7)	14 (10.6)
Investigational drug	10 (7.6)	10 (7.6)
Atomoxetine hydrochloride	8 (6.1)	7 (5.3)
Clonidine	7 (5.3)	5 (3.8)
Concomitant ADHD medications used by ≥2% of participants (either treatment arm), *n* (%)		
Lisdexamfetamine	7 (5.3)	9 (6.8)
Immediate-release MAS	9 (6.9)	8 (6.1)
Methylphenidate	1 (0.8)	6 (4.5)

ADHD, attention-deficit/hyperactivity disorder; MAS, mixed amphetamine salts.
