# Supplementary material for: SHP465 Mixed Amphetamine Salts in the Treatment of Attention-Deficit/Hyperactivity Disorder in Children and Adolescents: Results of a Randomized, Double-Blind Placebo-Controlled Study
Source: J Child Adolesc Psychopharmacol. 2018 Oct 20;28(1):19–28. doi: 10.1089/cap.2017.0053 (PMC5771539; doi:10.1089/cap.2017.0053)
Supplement: Supplemental data [file Supp_Table2.pdf]

Supplementary Table S2. Summary of Efficacy Findings, Full Analysis Set

	Placebo		*SHP465 MAS*
ADHD-RS-IV total score			
Mean ± SD score at baseline*	40.0 ± 6.96		39.0 ± 6.95
Mean ± SD score at week 4†	28.2 ± 13.71		17.3 ± 11.78
LS mean (95% CI) change at week 4†	−10.8 (−13.0, −8.5)		−20.7 (−22.9, −18.5)
LS mean (95% CI) treatment difference at week 4†		−9.9 (−13.0, −6.8)	
p-Value (degrees of freedom, *t*-statistic)§		<0.001 (241, −6.23)	
Effect size¢		0.80	
ADHD-RS-IV hyperactivity/impulsivity subscale score			
Mean ± SD score at baseline*	17.9 ± 5.52		17.0 ± 5.86
Mean ± SD score at week 4†	12.8 ± 7.71		7.1 ± 6.24
LS mean (95% CI) change at week 4†	−4.5 (−5.6, −3.4)		−9.5 (−10.6, −8.4)
LS mean (95% CI) treatment difference at week 4†		−4.9 (−6.5, −3.4)	
Nominal *p*-value (degrees of freedom, *t*-statistic)§		<0.001 (241, −6.25)	
Effect size¢		0.81	
ADHD-RS-IV inattentiveness subscale score			
Mean ± SD score at baseline*	22.1 ± 3.57		22.0 ± 3.20
Mean ± SD score at week 4†	15.4 ± 7.33		10.2 ± 6.52
LS mean (95% CI) change at week 4†	−6.2 (−7.5, −5.0)		−11.2 (−12.5, −10.0)
LS mean (95% CI) treatment difference at week 4†		−5.0 (−6.7, −3.2)	
Nominal *p*-value (degrees of freedom, *t*-statistic)§		<0.001 (239, −5.62)	
Effect size¢		0.72	
CGI-I score			
Mean ± SD score at week 1*	3.5 ± 0.82		2.9 ± 1.08
Mean ± SD score at week 4‡	2.9 ± 1.20		2.1 ± 1.13
LS mean (95% CI) at week 4‡	3.0 (2.8, 3.2)		2.2 (2.0, 2.4)
LS mean (95% CI) treatment difference at week 4‡		−0.8 (−1.1, −0.5)	
p-Value (degrees of freedom, *t*-statistic)§		<0.001 (242, −5.10)	
Effect size¢		0.65	
CGI-I*			
Improved£ at final on-treatment assessment, *n* (%)	47 (36.4)		77 (60.2)
Nominal *p*-value (degrees of freedom, CMH statistic)‖		<0.001 (1, 12.7799)	

*Based on *n* = 129 for placebo and *n* = 128 for SHP465 MAS.

†Based on *n* = 117 for placebo and *n* = 113 for SHP465 MAS.

‡Based on *n* = 117 for placebo and *n* = 112 for SHP465 MAS.

§Based on LS mean treatment difference from placebo; mixed-effects model for repeated measures analysis included treatment group, visit, age group, interaction of the treatment group × visit as factors, baseline score as a covariate, and an adjustment for the interaction of the baseline score × visit.

¢The difference in LS means divided by the estimated standard deviation from the unstructured covariance matrix.

£Improved categorized as scores of 1 (very much improved) and 2 (much improved).

‖Based on the Cochran-Mantel-Haenszel test stratified by age group and baseline CGI-Severity score.

ADHD, attention-deficit/hyperactivity disorder; ADHD-RS-IV, ADHD Rating Scale IV; CGI-I, Clinical Global Impressions-Improvement; CI, confidence interval; CMH, Cochran-Mantel-Haenszel; FAS, full analysis set; LS, least squares; MAS, mixed amphetamine salts; SD, standard deviation.
